# A Pilot Study: Changes of Intestinal Microbiota of Patients With Non-small Cell Lung Cancer in Response to Osimertinib Therapy

**DOI:** 10.3389/fmicb.2020.583525

**Published:** 2020-11-10

**Authors:** Jing Cong, Yuguang Zhang, Yadong Xue, Chuantao Zhang, Mingjin Xu, Dong Liu, Ruiyan Zhang, Hua Zhu

**Affiliations:** ^1^College of Marine Science and Biological Engineering, Qingdao University of Science and Technology, Qingdao, China; ^2^Key Laboratory of Forest Ecology, Environment of State Forestry Administration, Institute of Forestry Ecology, Environment and Protection, Chinese Academy of Forestry, Beijing, China; ^3^Department of Oncology, The Affiliated Hospital of Qingdao University, Qingdao University, Qingdao, China; ^4^Department of Radiotherapy, Qingdao Central Hospital, Qingdao, China

**Keywords:** intestinal microbiota, 16S rRNA sequencing, non-small cell lung cancer patients, osimertinib therapy, ecological network analysis

## Abstract

Osimertinib contributes to the higher efficacy and few intestinal side effects in non-small cell lung cancer (NSCLC) patients with T790M mutation. Previous studies has reported that intestinal microbiota play important roles in drug efficacy and toxicity. However, we have known less about the changes of intestinal microbiota in response to osimertinib therapy. In this pilot study, we used longitudinal sampling with 6 weeks sampling collection intervals for about 1 year to model intestinal microbial changes based on the 16S rRNA genes sequencing in fecal samples from NSCLC patients in response to osimertinib therapy. The results showed that there was no significantly different on the intestinal microbial composition at the phylum, family, and genus level among NSCLC patients with different treatment cycles (*P* > 0.05). There were no significant differences in alpha diversity characterized by the richness, Shannon diversity, and phylogenetic diversity based on the Welch’s *t*-test among NSCLC patients in response to osimertinib therapy (*P* > 0.05). However, the dissimilarity test and principal coordination analysis showed a few differences among NSCLC patients. The intestinal microbial markers were changed in post-therapy (*Sutterella*, *Peptoniphilus*, and *Anaeroglobus*) compared to that in pre-therapy (*Clostridium XIVa*). Furthermore, the phylogenetic molecular ecological networks (MENs) were influenced by osimertinib therapy based on the module number, link number, and module taxa composition of the first six groups. Overall, it indicated that osimertinib therapy changed the intestinal microbiota to some extent, though not completely. In all, this pilot study provides an understanding of changes of intestinal microbiota from NSCLC patients in response to osimertinib therapy. No complete changes in intestinal microbiota seem to be closely linked with the few intestinal side effects and higher efficacy in response to osimertinib therapy.

## Introduction

Lung cancer remains the leading cause of cancer-related deaths worldwide (Torre et al., 2016). Non-small cell lung cancer (NSCLC) accounts for most of all cases of lung cancer, including adenocarcinoma, squamous cell carcinoma, and large-cell lung cancer, which is generally diagnosed at a terminal stage of lung cancer. For a long time, platinum-based chemotherapy has represented the cornerstone for the first-line treatment of advanced NSCLC patients (Santarpia et al., 2017a), although with several limitations, including a number of side effects and a dismal overall survival. In recent years, the development of specific molecularly targeted agents has primarily changed the therapeutic landscape for advanced NSCLC patients, including epidermal growth factor receptor-tyrosine kinase inhibitors (EGFR-TKIs)-, anaplastic lymphoma kinase (ALK)-, and BRAF-inhibitors (Rosell and Karachaliou, 2016). These therapies have greatly improved the survival and quality of NSCLC patients. Gefitinib, afatinib, and erlotinib are the standard first-line treatment for advanced EGFR mutated NSCLC patients. After a variable length of time from starting treatment, the resistance mechanisms of first- and second- generation EGFR-TKIs inevitably emerge. The T790M mutation at exon 20 within the kinase domain of EGFR is the most common mechanism of acquired resistance, which occurs in approximately 50–60% of EGFR-TKI-resistant tumors.

Osimertinib is the third-generation for the treatment of patients with metastatic EGFR T790M-positive NSCLC (Cross et al., 2014), which is the first compound granted US Food and Drug Administration (FDA) and European Medicine Agency (EMA) approval (Santarpia et al., 2017b). Soria et al. (2018) found that the advanced NSCLC patients with previously untreated, EGFR mutation-positive receiving osimertinib had the significantly longer median progression-free survival (PFS) than those receiving gefitinib or erlotinib in a double-blind, phase 3 trial (18.9 vs. 10.2 months; *P* < 0.001). The median overall survival (OS) was 38.6 months in response to osimertinib therapy and 31.8 months in response to gefitinib or erlotinib therapy (Ramalingam et al., 2020). Furthermore, there were less adverse events of grade 3 or higher in the osimertinib group than that in the comparator group (34 vs. 45%) (Soria et al., 2018). Mok et al. (2017) reported that the median duration of PFS in these T790M-positive advanced NSCLC patients with osimertinib, who had disease progression after first-line EGFR-TKI therapy, was significantly longer than those with platinum therapy plus pemetrexed (10.1 vs. 4.4 months; *P* < 0.001). The less adverse events of grade 3 or higher were lower with osimertinib compared to the platinum therapy plus pemetrexed (23 vs. 47%) (Mok et al., 2017). Based on its significant efficacy, safety and favorable toxicity profile, osimertinib has been considered as a therapeutic option preferable to early generation EGFR-TKI for further improving the clinical outcome of EGFR-mutated patients (Mok et al., 2017). However, the disease would progress after receiving osimertinib therapy for approximately 10 months. Thus, some novel therapeutic strategies should overcome the osimertinib resistance.

Recently, intestinal microbiota has emerged as an “organ” that plays a key role in health and disease. The intestinal microbial composition shows high inter-individual variations (Huttenhower et al., 2012). Various studies have proved that effect of drug intake in intestinal microbiota (Wu et al., 2017). In turn, intestinal microbiota can also contribute to the different in response to a specific drug in different individuals (Wu et al., 2017). The intestinal microbiota can directly transform the drug or change the host’s metabolism and immune system to modify the pharmacodynamics of a medication (Rajpoot et al., 2018). Therefore, understanding the role of intestinal microbiota in drug response may contribute to the development of microbiome-targeting approaches that improve the drug efficacy.

Previous studies has reported that the intestinal microbiota play important roles in drug efficacy and toxicity in response to chemotherapeutic drugs (Alexander et al., 2017; Cong et al., 2019). These studies showed that drugs could change the composition of intestinal microbiota for patients. However, these works drew the conclusion with only a few times points. The dynamic patterns of microbial communities across longer time scales with drug usage remain unclear. In this study, we longitudinally tracked the changes of intestinal microbiota in NSCLC patients in response to targeted drug osimertinib therapy for nine cycles based on the 16S rRNA sequencing data. This pilot study will help the development of personalized medicine, and try to modulate the intestinal microbiota to manage drug efficiency on the level of the individual (Doestzada et al., 2018).

## Materials and Methods

### Study Subjects and Microbial Sampling

#### Non-small Cell Lung Cancer (NSCLC) Patients

**FIGURE 1:**
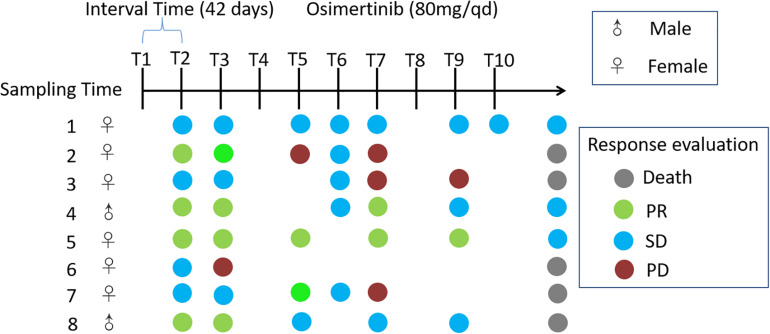
Timeline representing the clinical course of eight patients. Key time points include timing of fecal sampling; sampling time was ranged from April 2017 to May 2018; Response evaluations include partial response (PR), stable disease (SD), progression disease (PD) and death. Response evaluations were due to December 2018. Daily dosage of osimertinib was administered orally as one 80 mg tablet.

Eight adults with locally advanced (stage IIIB) or metastatic (stage IV) NSCLC with confirmed T790M mutation from the Affiliated Hospital of Qingdao University, who have received prior EGFR-TKI therapy, were recruited to the pilot study (Figure 1). Exclusion criteria included that those who have inflammatory bowel disease, irritable bowel syndrome, and other intestinal diseases, and were treated with antibiotics or probiotics usage, during the sampling. Sampling time was ranged from April 2017 to May 2018. Daily dosage of osimertinib was administered orally as one 80 mg tablet. Total of 65 fecal samples were collected from nine cycles of osimertinib therapy. The sample collection intervals of every cycle were about 6 weeks. Fecal samples were self-sampled in the morning prior to the start of drug usage, including that before the first therapy, before the second therapy, before the third therapy, before the fourth therapy, before the fifth therapy, before the sixth therapy, before the seventh therapy, before the eighth therapy, before the ninth therapy, and before the tenth therapy, named by T1, T2, T3, T4, T5, T6, T7, T8, T9, and T10, respectively.

#### Healthy Individuals

Control samples were obtained from 21 healthy individuals. These healthy individuals, who have any recorded antibiotics or probiotics usage, and gastrointestinal tract disorders within 1 month preceding the sample collection, were excluded. The collected samples from the healthy individuals were named by H.

All of the study subjects have been local residents of Qingdao city. This pilot study was approved by the Affiliated Hospital of Qingdao University Institutional Review Board, and all pilot study subjects signed the informed consent before participation. Fresh fecal samples were put into 5 ml tubes and immediately stored at −80°C until the day of analysis.

### DNA Extraction, PCR Amplification of 16S rRNA Gene, Amplicon Sequencing and Data Processing

Total genomic DNA was extracted using the DNA Stool Kit from Tiangen (Wu et al., 2016) and purity were monitored on 1% agarose gels. 16S rRNA gene of V3–V4 regions amplicon sequencing was carried out employing the 16S Metagenomic Sequencing Library Preparation protocol developed by Illumina (San Diego, California, United States) using the bacterial universal primers (357F-806R) (Cong et al., 2018). The PCR amplification products were purified with Qiagen Gel Extraction Kit (Qiagen, Germany). The DNA quality was assessed on the Qubit^®^ 2.0 Fluorometer (Thermo Fisher Scientific) and Agilent Bioanalyzer 2100 system. Finally, bacterial DNA amplicons were sequenced from each fecal sample for 2 × 250 bp paired-end sequencing based on the Illumina Hiseq 2500.

### 16S rRNA Amplicon Sequencing Data Analysis

Raw sequences were separated into samples by barcodes based on the Galaxy Illumina sequencing pipeline. Ambiguous, adapters, and low-quality reads (“N”) were trimmed by Btrim (Kong, 2011). Forward and reverse reads were incorporated into a whole sequence by FLASH (Magoc and Salzberg, 2011). After quality control of the raw data, the clean reads were clustered into operational taxonomic units (OTUs) by using UCLUST at 97% similarity level (Edgar, 2010). Each OTU was considered to represent a species (Deng et al., 2012). The ribosomal database project (RDP) classifier was used to determine the taxonomic assignment (Wang et al., 2007). Rarefaction analysis was performed using the original detected OTUs.

### Network Analysis

Intestinal microbial ecological networks were constructed and analyzed by random matrix theory (RMT) methods based on the online MENA pipeline. OTUs detected in more than half in each group were used to ensure reliable correlations. To compare with different networks, the same cutoff of 0.85 was applied to construct ecological networks for intestinal microbial communities. Each network was divided into modules by the fast greedy modularity optimization to describe the modularity property. In addition, a network developed by OTU abundance data represented the ecological links of different OTU nodes (OTUs) in a microbial community, and different nodes played distinct roles (Guimerà et al., 2007).

### Statistical Analysis

The Shannon index, richness, and phylogenetic diversity were calculated for alpha diversity analysis, which presented complexity of species diversity for samples. The different tests of alpha diversity for different groups were performed by Wilcoxon Rank Sum Test. Beta diversity was calculated by Bray-Curtis distance. Differences in beta diversity were identified using the multiple response permutation procedure (MRPP) algorithm. Community structure based on beta diversity was visualized using principal coordinate analysis (PCoA). Linear Discriminant Analysis with Effect Size (LEfSe) was used to identify the significant *P*-values associated with microbial clades and functions. Characteristics with a LDA score cutoff of 2.0 were known as being different. Significantly different biomarkers at the phylum and genus levels were identified using STAMP (v2.1.3). An absolute Pearson’s correlation was based on a significance level under 0.05. Principal components analysis (PCA) was used to determine the changes of intestinal microbiota based on significant different genera and OTUs. The R software package (v3.4.1) was used for all statistical analysis, except for two-tailed unpaired *t*-tests and Pearson correlation by IBM SPSS statistic 19.0 to determine the significance of the differences.

## Results

### Study General Characteristic

NSCLC patients and control subjects were matched for age, sex as well as body mass index (BMI) in this study (Table 1). Eight NSCLC patients with locally advanced (stage IIIB) or metastatic (stage IV) NSCLC treated with osimertinib therapy after progression were enrolled in the present study. Response evaluation was administered every 6 weeks. During the treatment, no antibiotics were applied. Patients were classified based on radiological evaluation according to Response Evaluation Criteria in Solid Tumors (RECIST 1.1) (Schwartz et al., 2016). Fecal samples were collected every 6 weeks during therapy until disease progression, or death, or the study self-withdrawal, according to the informed consent and study protocol. Dynamic variation of intestinal bacterial characteristics was evaluated and analyzed by metagenomic sequencing.

**TABLE 1:** Characteristics of the study subjects and the samples.

Subject group	No. of subjects (male/female)	Mean age (range)	Mean BMI (range)	Treatment period (sample number)									
				
				T1	T2	T3	T4	T5	T6	T7	T8	T9	T10
Healthy individuals (H)	21 (4/17)	54 (26–64)	23.4	21									
Non-small cell lung cancer patients (T)	8 (2/6)	60 (52–68)	24.4	8	8	8	7	7	7	6	5	5	4

### Changes of Intestinal Microbial Composition at the Taxonomical Level From NSCLC Patients in Response to Osimertinib Therapy

A total of 678 OTUs were defined with RDP annotations, including 351 OTUs belonging to 109 genera, and 327 OTUs of unclassified genera. Rarefaction curves showed that most samples leveled out between 100 and 250 taxa (Supplementary Figure S1). At the phylum level, the distribution pattern of the top six phylotypes (comprising about 99% of the total counts) in each group is shown in Supplementary Figure S2. We explored the differences of phylum Bacteroidetes, Firmicutes, Proteobacteria, Verrucomicrobia, Actinobacteria, and Fusobacteria in NSCLC patients and healthy individuals (Supplementary Table S1). The NSCLC samples showed no obvious differences in relative abundance of these phylotypes between pre-therapy and post-therapy (*P* > 0.05). There were also no significant different between NSCLC samples and healthy samples (*P* > 0.05). At the family level, Bacteroidaceae, Lachnospiraceae, and Prevotellaceae were the top three family almost in NSCLC patients and healthy individuals (Supplementary Figure S3). There were almost no significant differences in relative abundance of selected taxa between pre-therapy samples and post-therapy samples, and between NSCLC samples and healthy samples (*P* > 0.05, Supplementary Table S2). At the genus level, *Bacteroides* was the most abundant genus, followed by *Prevotella* in both NSCLC patients and healthy individuals, except for T9 and T10 (Supplementary Figure S4). We also explored the differences of the genus *Bacteroides*, *Prevotella, Faecalibacterium*, and other sixteen genera in NSCLC patients in response to osimertinib therapy. The results also showed that no significant differences were detected between pre- and post-therapy samples, and between NSCLC patients and healthy individuals (*P* > 0.05, Supplementary Table S3).

### Changes of Intestinal Microbial Diversity From NSCLC Patients in Response to Osimertinib Therapy

**FIGURE 2:**
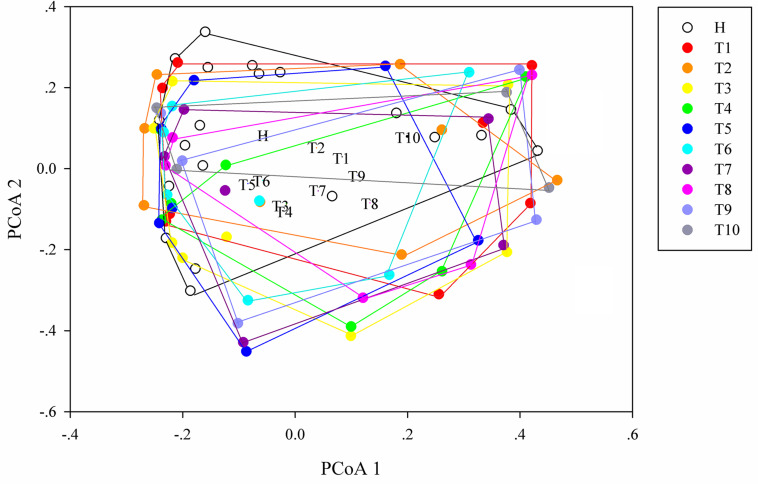
Principal coordinates analysis (PCoA) ordination (operational taxonomic units = 97% 16S rRNA sequence similarity) showing different microbial composition between NSCLC patients and healthy individuals based on the Bray-Curtis dissimilarity matrix.

As measures of alpha diversity (Supplementary Figure S5), which describes diversity within each sample, we used richness (number of distinct species present in samples), phylogenetic diversity, and Shannon diversity to explore the changes in eight patients in response to osimertinib therapy. The results showed that alpha diversity of individuals changed greatly (Supplementary Figure S6). Most samples in group T showed less species richness, phylogenetic diversity, and Shannon diversity than those in group H (Table 2). The Welch’s *t*-test showed almost no significant differences between group H and group T, and between pre-therapy and post-therapy (*P* > 0.05, Supplementary Table S4). Dissimilarity analysis showed significant differences between pre-therapy (T1) and post-therapy (T2, T3, T4, T5, T6, T7, T8, T9, T10) based on the MRPP (*P* < 0.05, Supplementary Table S5). However, no significant differences in group H and group T (*P* > 0.05, Supplementary Table S5). Principal coordination analysis based on Bray-curtis dissimilarity index showed a little separation between healthy individuals and NSCLC patients (Figure 2).

**TABLE 2:** The richness, phylogenetic diversity, and Shannon diversity in non-small cell lung cancer patients and healthy individuals.

Group	Richness	Phylogenetic diversity	Shannon diversity
H	161 ± 39	12.25 ± 2.18	4.44 ± 0.83
T1	125 ± 39	9.84 ± 2.65	3.97 ± 0.96
T2	136 ± 56	10.87 ± 3.34	3.63 ± 1.82
T3	127 ± 55	9.81 ± 3.48	4.20 ± 0.85
T4	142 ± 47	11.01 ± 3.45	4.45 ± 0.83
T5	153 ± 41	11.52 ± 2.49	4.72 ± 0.53
T6	148 ± 61	11.61 ± 3.63	4.30 ± 1.34
T7	127 ± 49	10.14 ± 3.32	4.10 ± 0.82
T8	132 ± 46	10.16 ± 2.73	4.15 ± 0.69
T9	123 ± 41	10.03 ± 2.42	3.78 ± 0.78
T10	127 ± 64	10.12 ± 4.19	3.67 ± 1.33

*H represents the healthy individuals; Tn (n = 1, 2, 3, 4, 5, 6, 7, 8, 9, and 10) represents the non-small cell lung cancer patients in different cycles of osimertinib therapy.*

### Differences of Intestinal Microbiota From NSCLC Patients in Response to Osimertinib Therapy

**FIGURE 3:**
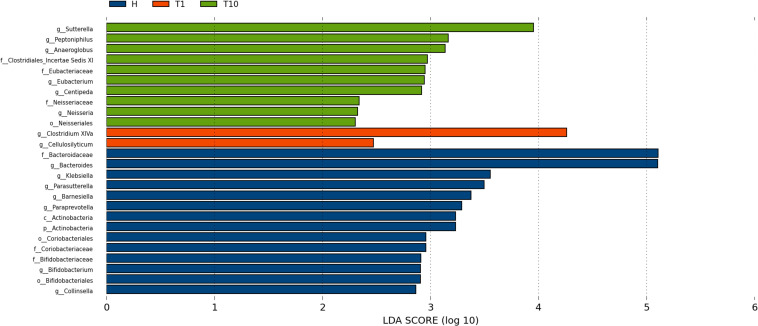
Microbial biomarkers among healthy individuals (H) and NLCLC patients (before the first therapy T1 and before the tenth therapy T10). Significantly different abundant taxa as biomarkers using Kruskal–Wallis test (*P <* 0.05) with LDA score > 2.0. based on LEfSe analysis.

**FIGURE 4:**
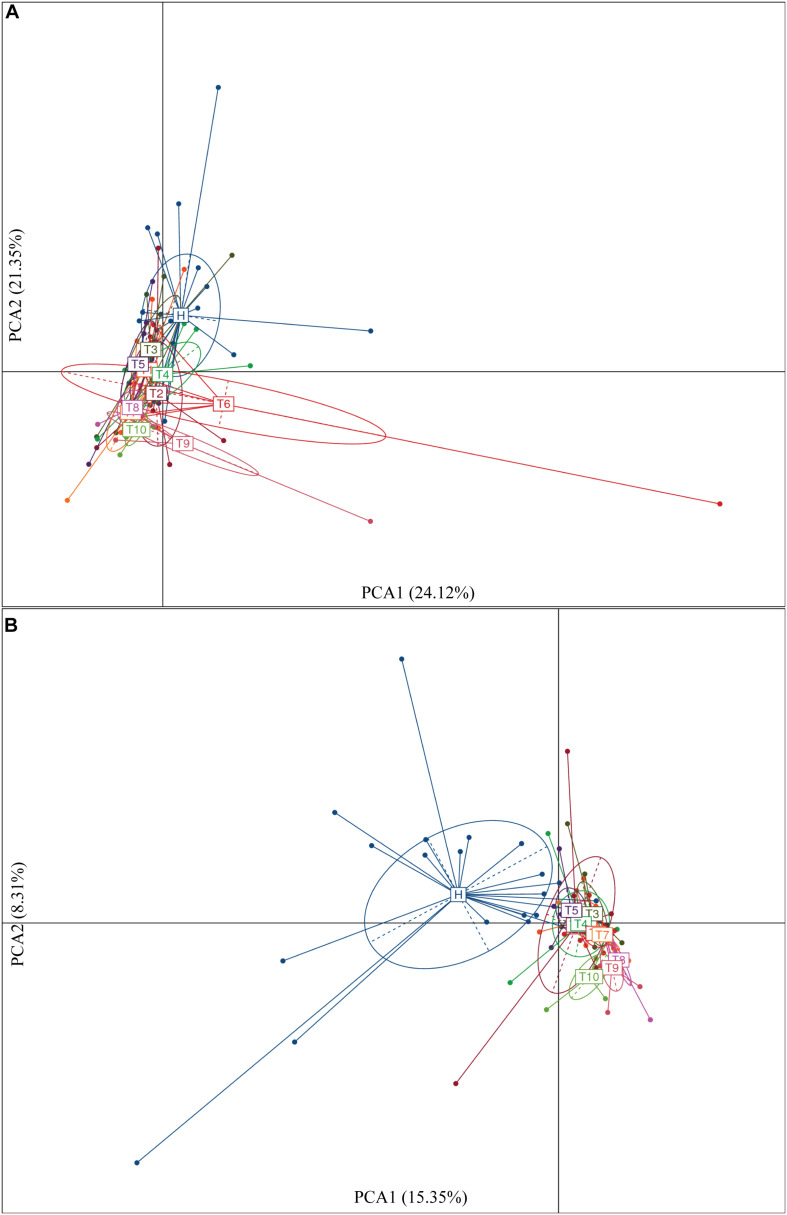
Significantly different genera **(A)** and OTUs **(B)** based on the PCA plot between healthy individuals and NSCLC patients in response to osimertinib therapy.

To identify intestinal microbial responses associated with osimertinib therapy at the taxonomical level, we determined microbial clade differences using LEfSe analysis (Figure 3). At the phylum level, we found that the higher proportions of Actinobacteria were observed in H than that in T1 and T10 (Supplementary Figure S7). At the genus level, greater proportions of *Bacteroides*, *Klebsiella*, and *Parasutterella* were detected in H than that in T1 and T10 (Supplementary Figure S7). The genera *Clostridium XIVa* and *Cellulosilyticum* were significantly enriched in T1 than that in H and T10 (Figure 3). The members of *Sutterella*, *Peptoniphilus*, *Anaeroglobus*, and *Neisseria* were more abundant in T10 than that in T1 and H (Figure 3). In addition, we constructed the PCA plot based on the significant different genera and OTUs in group T and group H (Figure 4). The results showed that the samples from NSCLC patients were well separated from the healthy individuals, but partly overlapped within different treatment cycles based on the different genera and OTUs (Figures 4A,B). We also selected the group T1, T3, T5, T7, and T10 to structure the PCA plot based on the significant distinct genera and OTUs (Supplementary Figure S8). It also showed the changes in response to osimertinib therapy.

### Molecular Ecological Network Analysis of Intestinal Microbiota From NSCLC Patients in Response to Osimertinib Therapy

**FIGURE 5:**
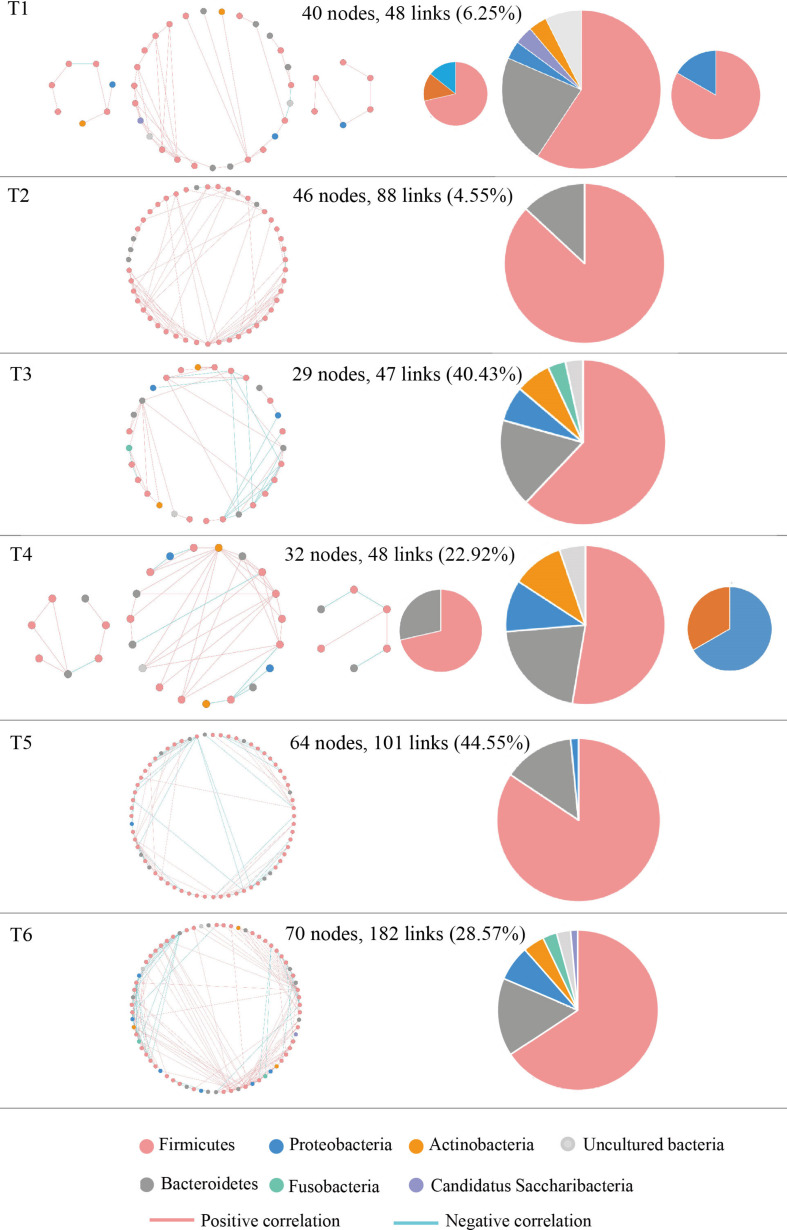
Highly connected modules with intestinal microbial networks of NSCLC patients in response to osimertinib therapy. Node colors represent different phyla; pie charts represent the composition of the modules. A blue link indicates a negative relationship between two phyla, whereas a pink indicates a positive relationship. The number in bracket means the ratio of negative links accounting for the total links.

The molecular ecological networks (MENs) were constructed for NSCLC patients to determine the effect of osimertinib therapy on microbial assemblages that potential interact with intestinal niches. We focused on representative networks from NSCLC patients with more than six biological duplications, including of the group T1, T2, T3, T4, T5, and T6. No less than five nodes to construct the modules in NSCLC samples (Figure 5). There were 3, 1, 1, 3, 1, and 1 module(s) in group T1, T2, T3, T4, T5, and T6 networks, respectively (Supplementary Table S6). Overall, taxa tended to co-occur (positive correlations, pink lines) rather than co-exclude (negative correlations, blue lines) (Figure 5). The negative correlations accounted for less than 45% of the potential interactions observed at each treatment stage (Figure 5). The negative correlations in NSCLC patients were increased by 22.32% from T1 to T6. The composition of the modules differed within each network and changed over the treatment time (Figure 5). Firmicutes almost dominated all the modules from each treatment stage in NSCLC patients. The phylum Fusobacteria presented in the modules before the third treatment (T3) and before the sixth treatment (T6). The phylum Fusobacteria was supposed to be more relevant to intestinal dysbiosis (Brennan and Garrett, 2019).

## Discussion

The identification of tyrosine kinase inhibitors (TKIs), has marked the advent of the era of precision medicine, which has revolutionized the diagnostic and therapeutic approach to NSCLC. Recently, osimertinib, which is designed to preferentially target sensitizing mutations and the T790M resistance mutation, over the wild-type receptor, has significantly improved survival and quality of life in molecularly defined subgroups of NSCLC patients. It has been reported that intestinal microbiome and drugs or drug metabolites interact with intestinal and systemic pharmacological effects. Intestinal microbiota play key roles in compound modifications including their activation (Tiago et al., 2014), inactivation (Haiser et al., 2013), or toxification (Wallace et al., 2010; Zimmermann and Zimmermann-Kogadeeva, 2019). In turn, the drug metabolite could change composition and structure. In this study, we explored that the changes of intestinal microbiota with NSCLC patients in response to osimertinib therapy for nine cycles.

Firstly, we examined the changes of intestinal microbial composition from NSCLC patients at the phylum and genus level in response to osimertinib therapy. Generally, the gut was dominated by members of four bacterial phyla, Firmicutes, Bacteroidetes, Proteobacteria, and Actinobacteria, with lesser and sporadic representation of other phyla, such as Fusobacteria and Verrucomicrobia (Rajilic-Stojanovic and De Vos, 2014; Budden et al., 2017; Wexler and Goodman, 2017). Consistent with it, our results demonstrated that the majority of all microbial populations identified in our participants were Bacteroidetes, Firmicutes, and Proteobacteria (Supplementary Figure S2). Previous numerous studies have characterized the lung microbiome using bronchoalveolar lavage microbiota of subjects with lung diseases. Significant differences are found in bacterial community composition between healthy and diseased lungs (Garzoni et al., 2013; Dickson et al., 2014). An increasing number of studies have revealed the close relationship between the intestinal microbial composition and lung diseases, known as the gut-lung axis (Budden et al., 2017; Zhuang et al., 2019). For example, an increase in the abundance of *Bacteroides fragilis* and total anaerobes, as well as a decrease in the relative abundance of *Faecalibacterium* spp., *Veillonella* spp., *Rothia* spp., and *Lachnospira* spp. in early life were associated with increased risk of asthma (Vael et al., 2008; Arrieta et al., 2015). Recently, Strickertsson et al. (2013), Amarnani and Rapose (2017), and Zhuang et al. (2019) found that patients with lung cancer in fecal microbiome showed elevated levels of Enterococcus, which could lead to increased DNA mismatch rate that indirectly promote rectal cancer. However, there were no significant differences in intestinal microbiota between NSCLC patients and healthy individuals and between pre-therapy and post-therapy at the phylum, family, and genus level in our pilot study (*P* > 0.05, Supplementary Tables S1–S3). It suggested that osimertinib therapy did not greatly change the relative abundance of intestinal microbiota in NSCLC patients based on the taxonomical level, and that intestinal microbiota of these NSCLC patients at the baseline did not differ more than that of healthy individuals.

Secondly, we explored the differences of alpha and beta diversity of intestinal microbiota from NSCLC patients in response to osimertinib therapy. In previous study, Zhuang et al. (2019) found that there was no significant reduction in alpha diversity of intestinal microbiota in lung cancer patients compared to healthy individuals. In line with it, our results indicated that there were almost no significant differences in richness, phylogenetic diversity, and Shannon diversity between NSCLC patients and healthy individuals (*P* > 0.05, Supplementary Table S4). Moreover, no significant difference was observed in the richness and Shannon diversity of intestinal microbiota in NSCLC patients between pre-therapy (T1) and post-therapy (T2, T3, T4, T5, T6, T7, T8, T9, T10) (*P* > 0.05, Supplementary Table S4), indicating that osimertinib therapy did not play great roles in alpha diversity of intestinal microbiota. However, we found that there was significantly different in beta diversity between pre-therapy and post-therapy in NSCLC patients based on the dissimilar test (*P* < 0.05, Supplementary Table S5), suggesting that osimertinib therapy has made the intestinal microbial community composition changed from the whole (Zhuang et al., 2019). At the same time, there was a little separation among NSCLC samples with different treatment cycles, and between healthy individuals and NSCLC patients (Figure 4). Previous studies reported that adaptive immunity in response to cancer therapy could shape the colonic microbiome (Scholz et al., 2014). We speculated that the differences probably due to the different immune status and dietary behavior among them in response to osimertinib therapy.

Thirdly, we found the variations of the intestinal microbial markers in NSCLC patients before the first treatment (T1) and after the ninth treatment (T10). The microbial biomarkers in healthy individuals were the *Bacteroides*, *Klebsiella*, and *Parasutterella*. *Bacteroides*, commonly found in the human intestine, has a symbiotic host-bacterial relationship with humans. They assist in digesting food and producing valuable nutrients and energy to meet the body needs. Some strains of *Klebsiella* are considered as a part of the normal flora of the human gastrointestinal tract. The genus of *Parasutterella* has been defined as a core component of human and mouse gut microbiota, and has been correlated with various health outcomes (Ju et al., 2019). These indicated that our healthy samples were eligible. The genus *Clostridium XIVa* was considered as the biomarker in T1, which produce butyrate and other short chain fatty acids, has been correlated with susceptibility to enteric pathogens (Lopetuso et al., 2013). It indicated that intestinal state in NSCLC patients was relatively healthy before the osimertinib therapy. The *Sutterella*, *Peptoniphilus*, and *Anaeroglobus* dominated in T10. *Sutterella* spp. has been associated with autism, gastrointestinal dysfunction and metabolic syndrome (Williams et al., 2012; Lavelle et al., 2015; Lim et al., 2017). *Peptoniphilus* are important causes of bloodstream infection (Brown et al., 2014). *Anaeroglobus* as an opportunistic pathogen was reported in clinical infection that presented as pneumonia with empyema (Wang et al., 2015). Changes of intestinal microbial markers between pre-therapy and post-therapy showed that osimertinib therapy had certain effects on the biomarker microbes, suggesting that there were probably underlying intestinal problems.

Finally, MENs of intestinal microbiota in NSCLC patients were also changed in response to osimertinib therapy. Microbes in the intestine are not independent individuals; however, they always make intricate and inter-connected ecological communities. The links between nodes (taxa) could explain the co-exclusion or co-occurrence correlations, mainly caused by the species performing exclusive and complementary functions (Zhou et al., 2011). The study results showed that the links in the module were distinctly increased from T1 to T6, suggesting that intestinal microbial interspecies interactions within the constructed ecological networks were changed, and the more complicated and compact of module was made in response to osimertinib therapy (Figure 5). Positive interactions usually signify that nodes cooperate with one another, while negative interactions indicate competition between the taxa (Deng et al., 2012). Violle et al. (2010) established the protist communities in laboratory microcosms to demonstrate that external disturbance accelerate microbial species-species competition. In our pilot study, the negative links increased distinctly from T1 to T6, suggesting that osimertinib probably played key roles in the competition relationships based on the species-species interactions of intestinal microbiota (Figure 5).

Although we followed the longitudinal sampling of these NSCLC patients for about 1 year, there are a number of limitations in the present study. Since only 8 patients were enrolled in our study, data of more participants are needed. Furthermore, we only collected stool samples on the basis of administration. We will carry out further study to collect stool samples on the basis of dose and duration of administration of the drug. In the future, the detail therapeutic modalities and clinical settings in targeting the “gut-lung axis” need to be paid more attention for solving NSCLC that seems promise. In addition, since the main research object of intestinal microbial diversity analysis is intestinal bacteria, the whole process of the experiment was carried out using bacterial universal primers for the amplification of bacterial marker genes; the virus and mycoplasma present in a small part were not analyzed. It is necessary to design a completion plan for this limitation in the future study.

## Conclusion

In conclusion, our pilot study found that osimertinib therapy changed intestinal microbial community composition from the whole, and made the intestinal microbial markers changed, as well as the varied microbial ecological networks for NSCLC patients. However, few roles were found in microbial composition changes at different taxonomical level and alpha diversity in response to osimertinib therapy. It indicated that osimertinib did not make radical change in intestinal microbiota of NSCLC patients. The partly changes of intestinal microbiota seem to be closely correlated with the few intestinal side effects and higher efficacy in these NSCLC patients with T790M mutation in response to osimertinib therapy.

## Data Availability Statement

The datasets presented in this study can be found in online repositories. The names of the repository/repositories and accession number(s) can be found in the article/Supplementary Material.

## Ethics Statement

The studies involving human participants were reviewed and approved by the Affiliated Hospital of Qingdao University Institutional Review Board. The patients/participants provided their written informed consent to participate in this study. Written informed consent was obtained from the individual(s) for the publication of any potentially identifiable images or data included in this article.

## Author Contributions

JC was involved in the conception and design of the study. JC, DL, CZ, MX, RZ, and HZ were involved in the collection and assembly of data. JC, YZ, and YX were involved in the data analysis and interpretation. All authors interpreted the data and wrote the manuscript and approved the final manuscript.

## Conflict of Interest

The authors declare that the research was conducted in the absence of any commercial or financial relationships that could be construed as a potential conflict of interest.
