# Supplementary material for: A Pilot Study: Changes of Intestinal Microbiota of Patients With Non-small Cell Lung Cancer in Response to Osimertinib Therapy
Source: Front Microbiol. 2020 Nov 10;11:583525. doi: 10.3389/fmicb.2020.583525 (PMC7683577; doi:10.3389/fmicb.2020.583525)
Supplement: Supplementary file 1 [file Table_1.DOCX]

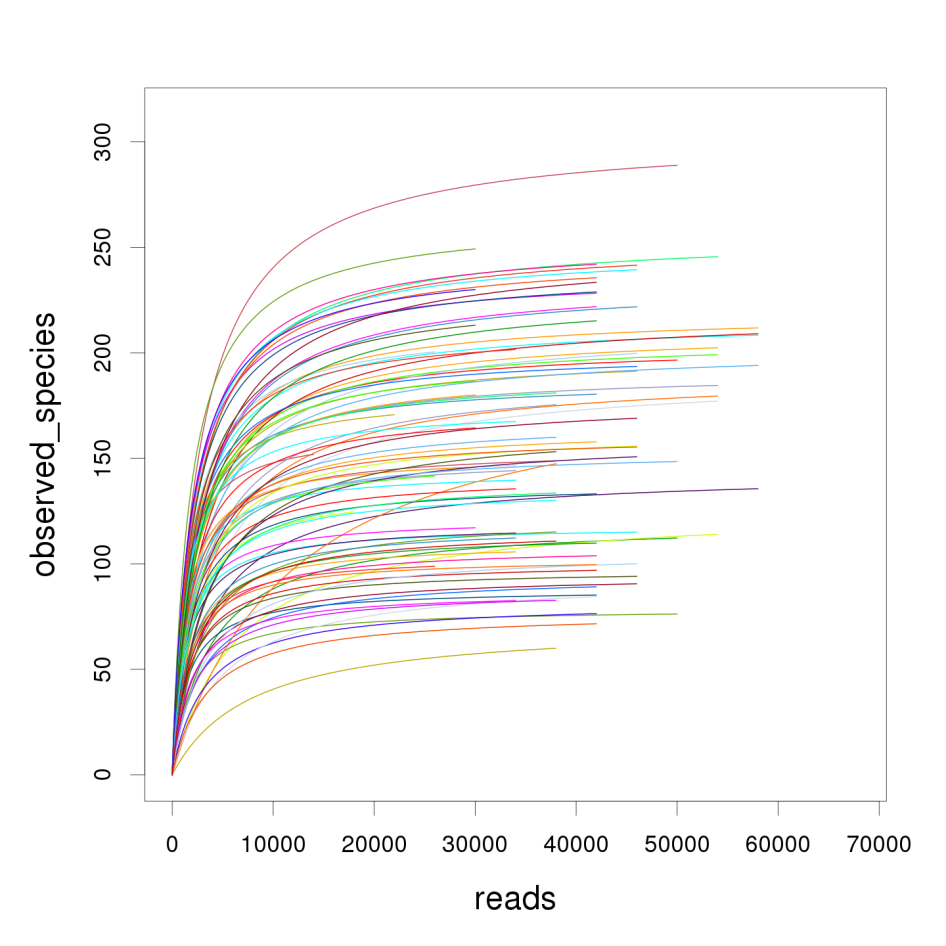


**Figure S1** Rarefaction curve for NSCLC patients and healthy individuals was generated at 97% similarity level.


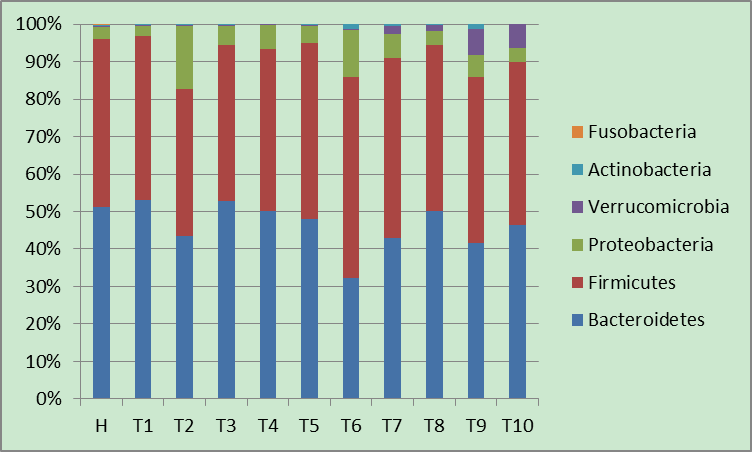


**Figure S2** The relative abundance of the top six phylotypes in NSCLC patients and healthy individuals.


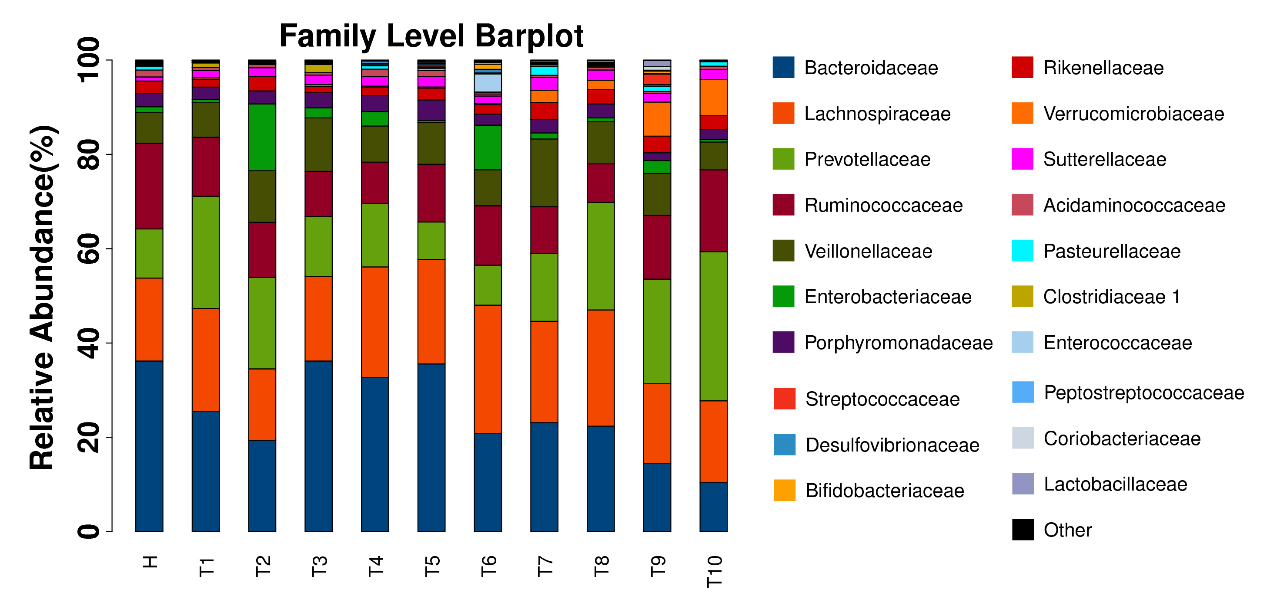
**Figure S3** The relative abundance of intestinal microbiota in NSCLC patients and healthy individuals at the family level.


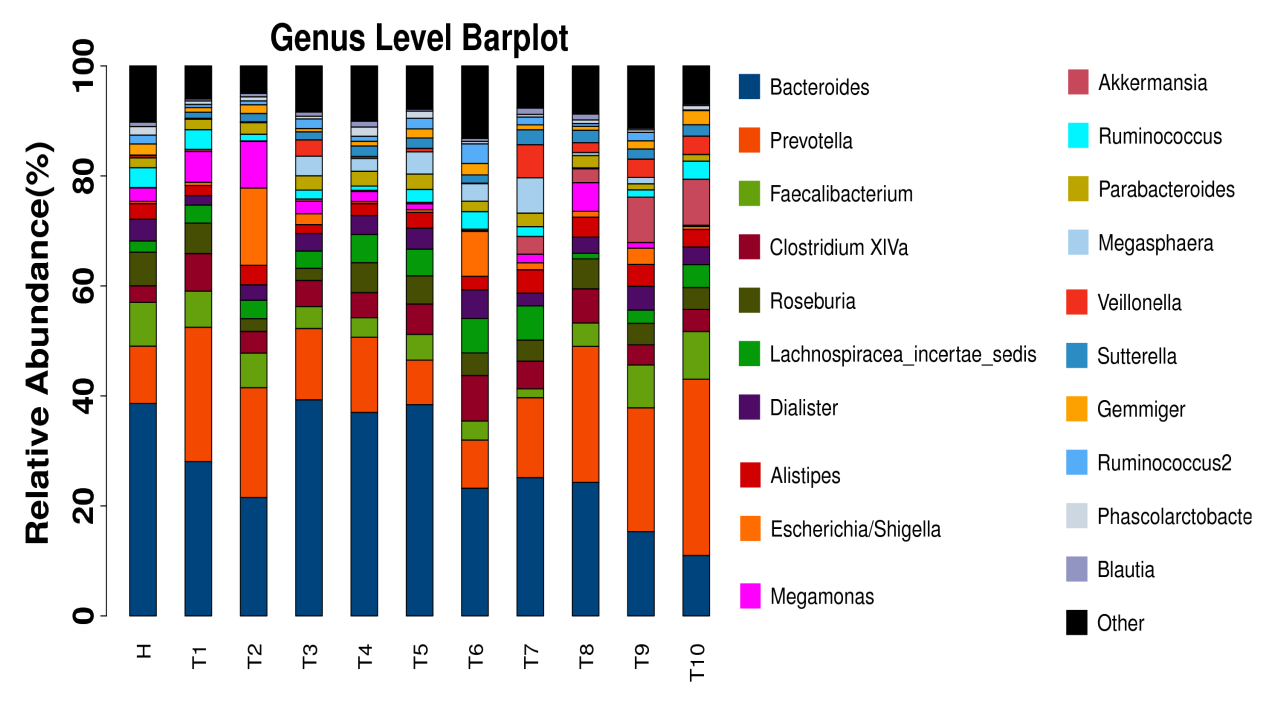


**Figure S4** The relative abundance of intestinal microbiota in NSCLC patients and healthy individuals at the genus level.


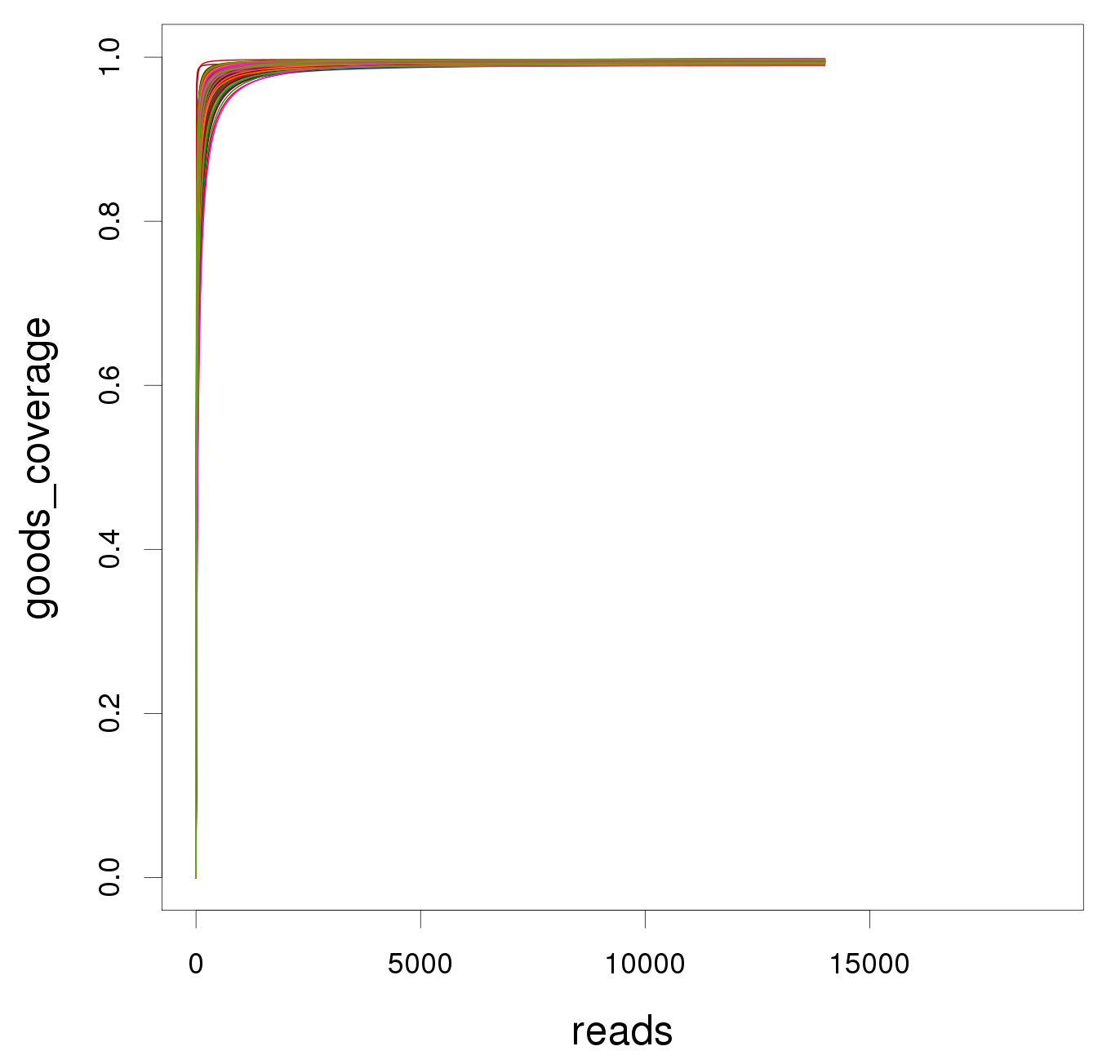


**Figure S5** The sequencing coverage of the detected intestinal samples in NSCLC patients and healthy individuals.


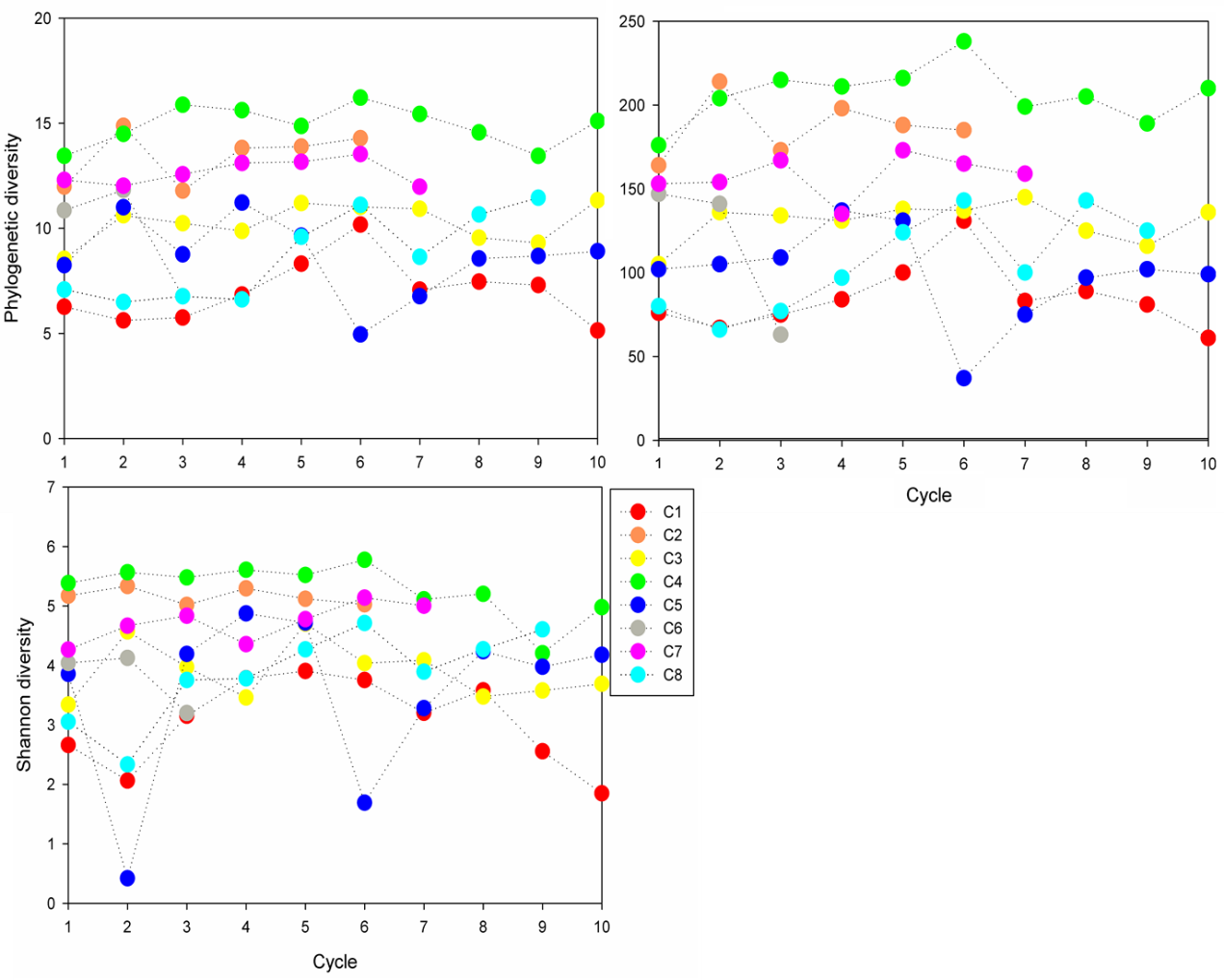


**Figure S6** Changes of the alpha diversity (richness, phylogenetic diversity and Shannon diversity) of NSCLC patients during osimertinib therapy. C1, C2, C3, C4, C5, C6, C7, C8, and C9 represent the 8 NSCLC patients, respectively.


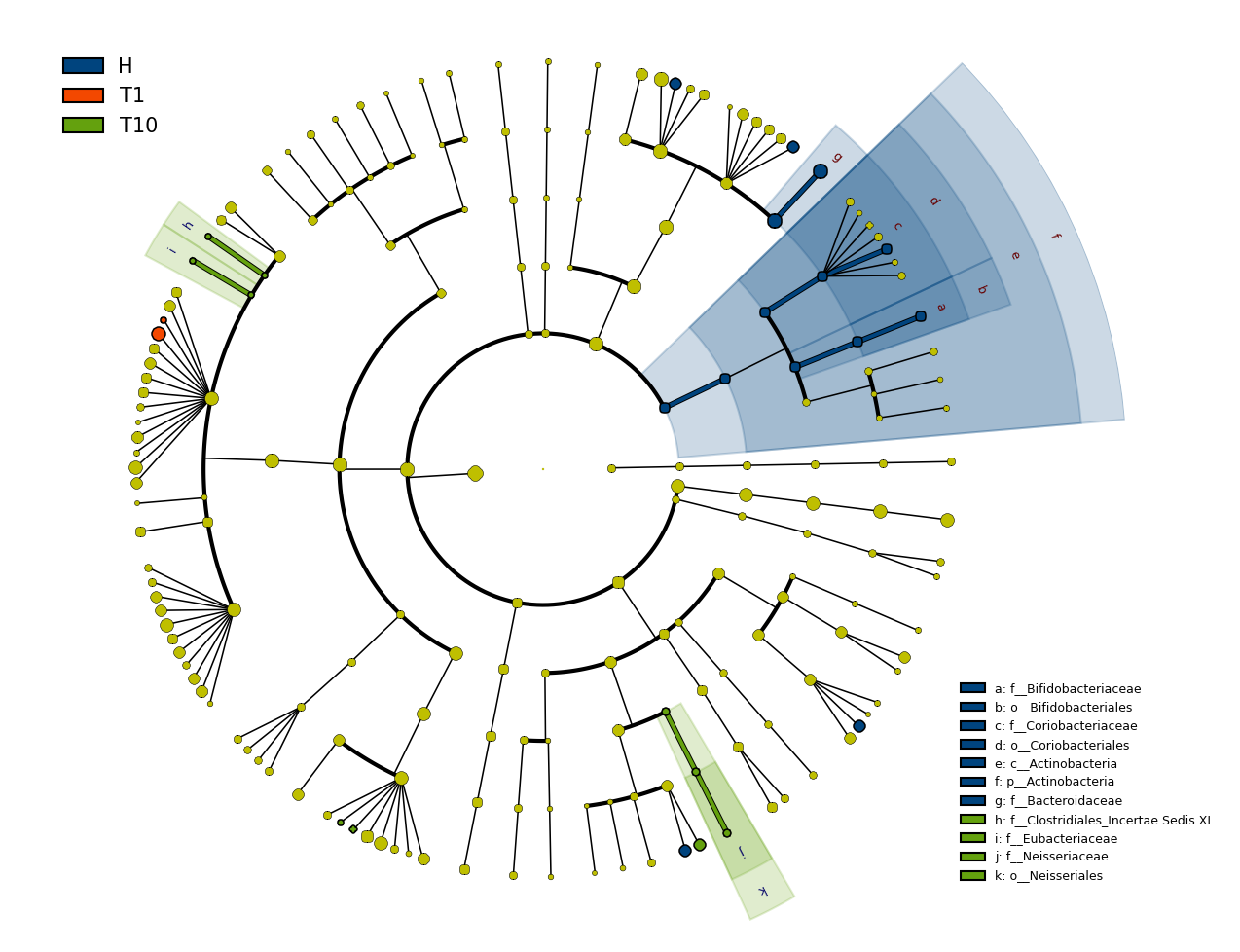


Figure S7 Cladogram represents the differentially abundant taxa in healthy individuals (H) and NLCLC patients (before the first therapy T1 and before the tenth therapy T10). The root of the cladogram represents the domain bacteria. The size of each node represents their relative abundance. Yellow represents no significantly different taxa. Significant differences are labeled by following the color of each group.


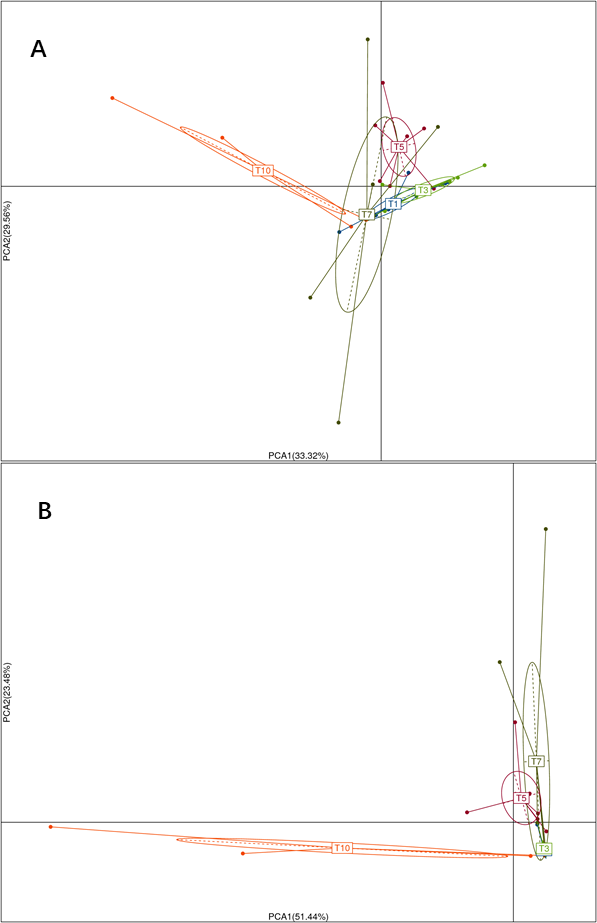


**Figure S8** PCA plot showing the significantly different genus (A) and OTUs (B) in different cycles of treatment with NSCLC patients
