# Supplementary material for: A Pilot Study: Changes of Intestinal Microbiota of Patients With Non-small Cell Lung Cancer in Response to Osimertinib Therapy
Source: Front Microbiol. 2020 Nov 10;11:583525. doi: 10.3389/fmicb.2020.583525 (PMC7683577; doi:10.3389/fmicb.2020.583525)
Supplement: Supplementary file 2 [file Table_2.DOCX]

**Table S1** The differences (*P* value) in the relative abundance of intestinal microbiota of NSCLC patients at the phylum level in response to the osimertinib therapy.

| Group | Bacteroidetes | Firmicutes | Proteobacteria | Verrucomicrobia | Actinobacteria | Fusobacteria |
| --- | --- | --- | --- | --- | --- | --- |
| H-T1 | 0.797 | 0.879 | 0.495 | 0.452 | 0.134 | 0.481 |
| T1-T2 | 0.347 | 0.664 | 0.259 | 0.440 | 0.444 | 0.325 |
| T1-T3 | 0.972 | 0.830 | 0.175 | 0.886 | 0.470 | 1.000 |
| T1-T4 | 0.723 | 0.945 | 0.135 | 0.658 | 0.435 | 0.162 |
| T1-T5 | 0.505 | 0.662 | 0.086 | 0.763 | 0.342 | 0.483 |
| T1-T6 | 0.052 | 0.273 | 0.258 | 0.729 | 0.239 | 0.347 |
| T1-T7 | 0.404 | 0.719 | 0.067 | 0.330 | 0.461 | 0.115 |
| T1-T8 | 0.818 | 0.964 | 0.288 | 0.320 | 0.639 | 0.207 |
| T1-T9 | 0.818 | 0.964 | 0.288 | 0.320 | 0.639 | 0.207 |
| T1-T10 | 0.672 | 0.995 | 0.273 | 0.188 | 0.152 | 0.506 |

**Table S2** The differences (*P* value) in the relative abundance of intestinal microbiota of NSLCLC patients at the family level in response to the osimertinib therapy

| Group | H-T1 | T1-T2 | T1-T3 | T1-T4 | T1-T5 | T1-T6 | T1-T7 | T1-T8 | T1-T9 | T1-T10 |
| --- | --- | --- | --- | --- | --- | --- | --- | --- | --- | --- |
| *Bacteroidaceae* | 0.198 | 0.413 | 0.144 | 0.279 | 0.104 | 0.474 | 0.757 | 0.704 | 0.135 | 0.070 |
| *Lachnospiraceae* | 0.394 | 0.246 | 0.532 | 0.793 | 0.938 | 0.437 | 0.969 | 0.769 | 0.539 | 0.611 |
| *Prevotellaceae* | 0.152 | 0.749 | 0.38 | 0.439 | 0.179 | 0.205 | 0.504 | 0.948 | 0.924 | 0.686 |
| *Ruminococcaceae* | 0.158 | 0.885 | 0.545 | 0.441 | 0.952 | 0.987 | 0.626 | 0.423 | 0.875 | 0.532 |
| *Veillonellaceae* | 0.817 | 0.703 | 0.582 | 0.96 | 0.810 | 0.961 | 0.377 | 0.809 | 0.812 | 0.842 |
| *Enterobacteriaceae* | 0.409 | 0.292 | 0.325 | 0.247 | 0.726 | 0.378 | 0.510 | 0.806 | 0.463 | 0.839 |
| *Porphyromonadaceae* | 0.875 | 0.956 | 0.744 | 0.676 | 0.455 | 0.824 | 0.907 | 0.862 | 0.519 | 0.759 |
| *Rikenellaceae* | 0.490 | 0.591 | 0.858 | 0.905 | 0.618 | 0.851 | 0.417 | 0.553 | 0.541 | 0.630 |
| *Sutterellaceae* | 0.212 | 0.794 | 0.623 | 0.575 | 0.507 | 0.942 | 0.498 | 0.524 | 0.772 | 0.586 |
| *Verrucomicrobiaceae* | 0.445 | 0.439 | 0.92 | 0.688 | 0.814 | 0.762 | 0.419 | 0.452 | 0.393 | 0.409 |
| *Acidaminococcaceae* | 0.302 | 0.897 | 0.836 | 0.423 | 0.488 | 0.763 | 0.840 | 0.992 | 0.819 | 0.855 |
| *Pasteurellaceae* | 0.234 | 0.364 | 0.745 | 0.277 | 0.238 | 0.322 | 0.177 | 0.313 | 0.348 | 0.394 |
| *Clostridiaceae* | 0.145 | 0.150 | 0.651 | 0.138 | 0.213 | 0.166 | 0.391 | 0.237 | 0.399 | 0.119 |
| *Enterococcaceae* | 0.411 | 0.351 | 0.351 | 0.356 | 0.230 | 0.356 | 0.363 | / | 0.266 | 0.391 |
| *Desulfovibrionaceae* | 0.633 | 0.635 | 0.996 | 0.581 | 0.207 | 0.074 | 0.867 | 0.474 | 0.941 | 0.858 |
| *Streptococcaceae* | **0.026** | 0.266 | 0.336 | 0.223 | 0.176 | 0.276 | 0.263 | 0.191 | 0.368 | 0.620 |
| *Bifidobacteriaceae* | 0.344 | 0.524 | 0.486 | 0.293 | 0.818 | 0.373 | 0.131 | 0.096 | 0.459 | 0.097 |
| *Peptostreptococcaceae* | 0.965 | 0.644 | 0.611 | 0.382 | 0.980 | 0.481 | 0.910 | 0.611 | 0.881 | 0.323 |
| *Coriobacteriaceae* | 0.254 | 0.375 | 0.826 | 0.703 | 0.246 | 0.381 | 0.400 | 0.318 | 0.384 | 0.705 |
| *Lactobacillaceae* | 0.880 | 0.943 | 0.444 | 0.236 | 0.302 | 0.367 | 0.579 | 0.386 | 0.378 | 0.390 |

**Table S3** The differences (*P* value) in the relative abundance of intestinal microbiota of NSLCLC patients at the genus level in response to the osimertinib therapy.

| Group | H-T1 | T1-T2 | T1-T3 | T1-T4 | T1-T5 | T1-T6 | T1-T7 | T1-T8 | T1-T9 | T1-T10 |
| --- | --- | --- | --- | --- | --- | --- | --- | --- | --- | --- |
| *Bacteroides* | 0.223 | 0.456 | 0.165 | 0.260 | 0.138 | 0.523 | 0.738 | 0.687 | 0.130 | 0.073 |
| *Prevotella* | 0.151 | 0.752 | 0.374 | 0.434 | 0.190 | 0.219 | 0.492 | 0.984 | 0.910 | 0.700 |
| *Faecalibacterium* | 0.604 | 0.928 | 0.364 | 0.268 | 0.498 | 0.293 | 0.091 | 0.511 | 0.835 | 0.709 |
| *Clostridium* | **0.035** | 0.115 | 0.282 | 0.242 | 0.529 | 0.775 | 0.345 | 0.772 | 0.141 | 0.219 |
| *Roseburia* | 0.796 | 0.175 | 0.163 | 0.971 | 0.876 | 0.622 | 0.568 | 0.983 | 0.636 | 0.652 |
| *Lachnospiracea* | 0.293 | 0.964 | 0.942 | 0.463 | 0.383 | 0.244 | 0.435 | 0.124 | 0.611 | 0.751 |
| *Dialister* | 0.192 | 0.525 | 0.324 | 0.386 | 0.190 | 0.073 | 0.657 | 0.437 | 0.145 | 0.407 |
| *Megamonas* | 0.409 | 0.767 | 0.537 | 0.498 | 0.412 | 0.321 | 0.499 | 0.951 | 0.484 | 0.467 |
| *Escherichia* | 0.568 | 0.297 | 0.341 | 0.644 | 0.810 | 0.329 | 0.521 | 0.612 | 0.301 | 0.911 |
| *Alistipes* | 0.503 | 0.568 | 0.906 | 0.874 | 0.614 | 0.803 | 0.389 | 0.519 | 0.535 | 0.647 |
| *Ruminococcus* | 0.989 | 0.354 | 0.434 | 0.293 | 0.652 | 0.905 | 0.537 | 0.284 | 0.483 | 0.937 |
| *Parabacteroides* | 0.894 | 0.847 | 0.605 | 0.549 | 0.466 | 0.992 | 0.651 | 0.786 | 0.485 | 0.594 |
| *Megasphaera* | 0.553 | 0.343 | 0.314 | 0.355 | 0.301 | 0.302 | 0.263 | 0.196 | 0.221 | 0.315 |
| *Sutterella* | 0.085 | 0.723 | 0.737 | 0.446 | 0.497 | 0.610 | 0.355 | 0.289 | 0.409 | 0.355 |
| *Gemmiger* | 0.346 | 0.532 | 0.619 | 0.943 | 0.129 | 0.188 | 0.893 | 0.828 | 0.333 | 0.246 |
| *Veillonella* | 0.544 | 0.445 | 0.267 | 0.569 | 0.390 | 0.568 | 0.261 | 0.221 | 0.198 | 0.185 |
| *Akkermansia* | 0.445 | 0.890 | 0.941 | 0.715 | 0.837 | 0.799 | 0.320 | 0.300 | 0.240 | 0.186 |
| *Phascolarctobacterium* | 0.309 | .155 | 0.821 | 0.409 | 0.491 | 0.761 | 0.822 | 0.999 | 0.806 | 0.866 |
| *Blautia* | 0.058 | .987 | 0.221 | 0.104 | 0.771 | 0.384 | 0.263 | 0.143 | 0.613 | 0.450 |

**Table S4** The differences (*P* value) between NSCLC patients and healthy individuals in alpha diversity of intestinal microbiota.

| Group | Richness | Phylogenetic diversity | Shannon diversity |
| --- | --- | --- | --- |
|  | *P* | | |
| H VS T1 | **0.036** | **0.018** | 0.204 |
| H VS T2 | 0.177 | 0.198 | 0.263 |
| H VS T3 | 0.068 | **0.031** | 0.495 |
| H VS T4 | 0.291 | 0.271 | 0.975 |
| H VS T5 | 0.631 | 0.464 | 0.417 |
| H VS T6 | 0.508 | 0.575 | 0.753 |
| H VS T7 | 0.083 | 0.074 | 0.378 |
| H VS T8 | 0.156 | 0.077 | 0.482 |
| H VS T9 | 0.06 | 0.056 | 0.121 |
| H VS T10 | 0.153 | 0.136 | 0.136 |
| T1 VS T2 | 0.669 | 0.507 | 0.651 |
| T1 VS T3 | 0.959 | 0.988 | 0.623 |
| T1 VS T4 | 0.474 | 0.470 | 0.324 |
| T1 VS T5 | 0.206 | 0.229 | 0.092 |
| T1 VS T6 | 0.403 | 0.295 | 0.585 |
| T1 VS T7 | 0.951 | 0.854 | 0.803 |
| T1 VS T8 | 0.793 | 0.838 | 0.723 |
| T1 VS T9 | 0.905 | 0.896 | 0.722 |
| T1 VS T10 | 0.970 | 0.889 | 0.664 |

**Table S5** The dissimilarity analysis in beta diversity of intestinal microbiota for NSCLC patients and healthy individuals.

| Group | MRPP | |
| --- | --- | --- |
|  | The group mean distance | *P* |
| H-T1 | 0.712 | 0.218 |
| H-T2 | 0.712 | 0.227 |
| H-T3 | 0.712 | 0.216 |
| H-T4 | 0.706 | 0.109 |
| H-T5 | 0.706 | 0.099 |
| H-T6 | 0.706 | 0.111 |
| H-T7 | 0.715 | 0.203 |
| H-T8 | 0.718 | 0.141 |
| H-T9 | 0.718 | 0.142 |
| H-T10 | 0.723 | 0.272 |
| T1-T2 | 0.564 | **0.004** |
| T1-T3 | 0.523 | **0.002** |
| T1-T4 | 0.052 | **0.003** |
| T1-T5 | 0.520 | **0.002** |
| T1-T6 | 0.520 | **0.004** |
| T1-T7 | 0.533 | **0.008** |
| T1-T8 | 0.564 | **0.006** |
| T1-T9 | 0.564 | **0.005** |
| T1-T10 | 0.564 | **0.008** |

**Table S6** Number of modules, nodes and links in the networks of NSCLC patients.

| Terms | Colorectal cancer patients | | | | | |
| --- | --- | --- | --- | --- | --- | --- |
|  | T1 | T2 | T3 | T4 | T5 | T6 |
| Sub-module | 3 | 1 | 1 | 3 | 1 | 1 |
| Nodes | 40 | 46 | 29 | 32 | 64 | 70 |
| Links (np/pp) | 48(3/45) | 88(4/84) | 47(19/28) | 48(11/37) | 101(45/56) | 182(52/130) |

Np: negative correlation, pp: positive correlation.
